# Different Impacts of COVID-19 on Quality of Therapy, Psychological Condition, and Work Life Among Occupational Therapists in Physical and Mental Health Fields

**DOI:** 10.3389/fpubh.2022.887069

**Published:** 2022-06-03

**Authors:** Daisuke Sawamura, Ayahito Ito, Hideki Miyaguchi, Haruki Nakamura, Toshiyuki Ishioka

**Affiliations:** ^1^Faculty of Health Sciences, Hokkaido University, Sapporo, Japan; ^2^Research Institute for Future Design, Kochi University of Technology, Kochi, Japan; ^3^Department of Human Behavior Science of Occupational Therapy, Graduate School of Biomedical and Health Sciences, Hiroshima University, Hiroshima, Japan; ^4^Japanese Association of Occupational Therapists, Tokyo, Japan; ^5^Department of Occupational Therapy, Saitama Prefectural University, Koshigaya, Japan

**Keywords:** COVID-19, occupational therapy, healthcare worker, therapy quality, psychological condition, work life, mental health

## Abstract

**Background:**

The negative impacts of the coronavirus disease 2019 (COVID-19) pandemic have worsened the quality of therapy, psychological condition, and work life of second-line healthcare workers and occupational therapists (OTs). However, no study has investigated whether the impact of COVID-19 varies among OTs working in different fields. This study aimed to investigate the differences on the impact of COVID-19 between OTs in the physical and mental health fields.

**Methods:**

A cross-sectional online survey was conducted in Japan between January 20 and January 25, 2021. A total of 4,418 registered OTs who were members of the Japanese Association of Occupational Therapists volunteered for this study. After screening using the exclusion criteria, 1,383 participants were classified into two groups based on their field (mental health and physical health), and their quality of therapy, psychological condition, and work life were analyzed.

**Results:**

OTs in the mental health field showed a greater decrease in therapy quality and increase in workload and a lower rate of decrease in working hours than those in the physical health field. In the multinomial logistic regression analysis, decreased and increased therapy quality and decreased therapy quality were significantly associated with depression in the physical health field, and decreased therapy quality was associated with insomnia in the mental health field. Furthermore, insomnia and anxiety were commonly associated with increased workload and working hours, respectively, in both fields, whereas anxiety and depression were associated with increased workload only in the physical health field.

**Conclusions:**

These results demonstrate that COVID-19 differently impacted quality of treatment, workload, work time, and psychological condition in the physical and mental health fields; moreover, the relationships among these are different in these two fields. These results highlight the importance of investigating the field-specific negative impacts of COVID-19 on OTs and may provide helpful information for devising tailored and effective prevention and intervention strategies to address these challenges.

## Introduction

The coronavirus disease 2019 (COVID-19) pandemic has had an unprecedented impact on society and led to a dramatic loss of human life worldwide, presenting a unique challenge to public health and socioeconomic welfare (1, 2). The repeated waves of COVID-19 outbreaks have resulted in social isolation (2), loss of accessibility (3), economic crises (4), substance abuse (5), and deterioration of the working environment (4, 6–8), which are reported to be closely related to the mental health of citizens and workers. In particular, many previous studies have reported on the relationship between the working environment and mental health, and significantly, concerns are increasing about the mental health, psychological adjustment, and recovery of healthcare workers treating and caring for patients with COVID-19 (9, 10). Several systematic reviews have revealed that frontline medical workers fighting the disease experience poor mental health, such as depression, anxiety, insomnia, and posttraumatic stress reactions (11–13). These negative impacts have also been reported among second-line healthcare professionals (6, 14, 15).

Occupational therapists (OTs) are healthcare workers who offer a broad variety of services to people of all age groups and are typically classified as second-line medical professionals who do not directly care for patients with COVID-19 during the acute phase (16). During the pandemic, occupational therapy has heightened the importance of enabling engagement in activities that provide meaning in life when participation in regular routines and activities is particularly challenging (17). However, contrary to this situation, their work life has changed due to the current pandemic, which has negatively affected their mental health (6, 16, 17). A global survey of individuals involved in the delivery of occupational therapy conducted by the World Federation of Occupational Therapists (WFOT) reported negative mental health impacts, overwork, and isolation in this group due to the COVID-19 pandemic and stated that practical support, reassurance, and prevention were vital to address these problems (17). In addition, for efficiency in work during the pandemic, respondents indicated that preparedness for ever-changing circumstances and needs was paramount. However, information on how such preparedness may be achieved is lacking.

Recently, although mental health problems have been associated with work-related stress, including long working hours and heavy workload on OTs, no study has investigated the differences in the impact of the COVID-19 pandemic on work life among OTs working in different fields. OTs work with all age groups in various fields of physical and psychosocial/mental health. They work in a wide variety of settings, including hospitals, clinics, daycare centers, rehabilitation centers, home care programs, special schools, industry (e.g., service industry, corporate sector), and the private sector, and the objectives and solutions required of OTs vary, depending on where they work (11). It is expected that the work changes and psychological impact of the recent pandemic will vary depending on the field in which they work, as previous studies of burnout syndrome among OTs reported a higher prevalence in the mental health field than in the physical health field (18, 19). Therefore, we focused on the differences in the negative impacts of the COVID-19 pandemic among occupational therapists between two representative fields from a macroscopic perspective: the mental health field and the physical health field.

OTs need to protect both clients and themselves from the COVID-19 virus when they undertake occupational therapy in hospitals. By avoiding closed spaces, crowded places, and closed-contact settings (3Cs), as proposed by the World Health Organization COVID-19 new normal guidelines, the WFOT has recommended telerehabilitation methods for providing treatment to clients. However, the introduction of telerehabilitation cannot be implemented uniformly due to differences in implementation methods such as group occupational therapy and one-on-one occupational therapy, as well as differences in clients' adaptability to new program delivery methods. If therapists and clients have no choice but to conduct the program in the same room, the degree of difficulty in conducting the program differs based on the client's understanding of infection prevention as well as of group and individual occupational therapy. In the physical health field, it is necessary to deal with the increased likelihood of therapists coming into physical contact with clients in the context of individual therapy. One is more likely to deal with programs that involve little body contact in group-based activities in the mental health field (20–22).

A group-based occupational therapy program is a necessary and appropriate intervention for exploring and developing distinct knowledge and skills, including basic social interaction skills, tools for self-regulation, goal setting, and learning and skills acquisition across the lifespan (23), and these benefits are often highlighted in the mental health field (24, 25). Moreover, previous studies have reported that patients with mental illnesses have a higher risk of COVID-19 infection and worsening mental illness because of their symptom characteristics (26, 27). Worsened mental health in these patients can lead to a burden on therapists and even deterioration of therapists' own mental health and consequent lower quality of therapy. Furthermore, previous studies have reported a higher prevalence of burnout syndrome caused by work-related stress in the mental health field than in the physical health field (18, 19). Therefore, it is expected that different impacts of COVID-19 on mental health problems and lower therapy quality are likely in these two fields.

This study aimed to investigate the differences in the impact of COVID-19 on work life, psychological condition, and work quality among OTs in two representative fields of occupational therapy: physical and mental health. Moreover, we sought to identify the relationship between psychological measurements and therapy quality in therapists during the pandemic in each field. Clarifying the differences in the impact of COVID-19 on work life among OTs between the two fields and the psychological effects underlying them can contribute to developing preventive and intervention strategies for predictive field-specific occupational problems in occupational therapy and devising solutions and initiatives for current issues in this field.

## Materials and Methods

### Research Protocol

A cross-sectional online survey was conducted in Japan from January 20 to 25, 2021, using Google Forms https://www.google.com/forms/about/. All respondents were occupational therapists who were members of the Japanese Association of Occupational Therapists, and an invitation for participation was sent to all registered members on January 20, 2021, *via* email.

The study protocol was approved by the Ethics Committee of Saitama Prefectural University (approval no. 20003) and was conducted in accordance with the latest version of the Declaration of Helsinki. Written informed consent was obtained from all the respondents, before and after answering the questionnaire.

### Online Questionnaire

#### Sociodemographic Characteristics

Participants were asked to complete a questionnaire on their sociodemographic characteristics, including age, sex, academic background, marital status (married or unmarried), history of psychiatric disorders (yes or no), employment type (full-time or part-time), managerial position (yes or no), and years of service.

#### Therapy Quality

Participants were asked to assess their own therapy quality and colleagues' therapy quality (increased, decreased, or unchanged) compared to the period before COVID-19.

#### Effects of the Pandemic on Work Life

Participants were required to answer items concerning their work situation, which included the acceptance of patients with COVID-19 at their workplace (“yes” or “no”); provision of information on COVID-19 by the workplace (7-point rating scale ranging from “1 = insufficient” to “7 = sufficient”); changes to working hours, workload, and homework compared to the period before COVID-19 (“increased,” “decreased,” or “unchanged”); and a free description item (fill-in-the-blank question).

#### Effects of the Pandemic on Daily Life

Participants were required to respond to the following items concerning daily life: efforts to avoid being infected (7-point rating scale ranging from “1 = never” to “7 = frequent”), efforts to not transmit the virus to others (same 7-point scale), frequency of contact with family (same 7-point scale), frequency of contact with friends (same 7-point scale), fewer outings (“yes” or “no”), attempts to avoid face-to-face conversations (yes or no), increased standard precautions at home (handwashing and gargling; yes or no), increased mask-wearing frequency (yes or no), increased social network sites usage (yes or no), and free description (fill-in-the-blank question).

### Psychological Measurement

Based on our previous study (6), we focused on differences in field-specific impacts on the psychological aspects of anxiety, depression, insomnia, and loneliness. To assess each psychological aspect, we used four validated questionnaires: the Zung Self-Rating Anxiety Scale (SAS) (28), Zung Self-Rating Depression Scale (SDS) (29), Japanese version of the Insomnia Severity Index (ISI–J) (30, 31) and Japanese version of the three-item loneliness scale (TILS) (32).

In this study, the cutoffs for detecting the presence of anxiety, depression, insomnia, and loneliness were set to 40 for the SAS (33), 50 for the SDS (34), 10 for the ISI–J (30, 31) and 6 for the TILS (32).

### Data Recruitment Process

To determine the eligibility of the data, exclusion criteria were set as follows: (1) history of psychiatric disorders; (2) inconsistent responses between “yes” or “no” questions and rating (e.g., “yes” to the change in outing frequency but rated the frequency as “unchanged”); (3) declaration that they do not regularly see clients; and (4) inconsistent answers on items about working hours. Finally, the sample data that fulfilled the following inclusion criteria were recruited for data analysis in this study: (1) OTs who work in the field of physical or mental health in medical facilities and (2) OTs who work full-time.

### Statistical Analysis

Analyses were conducted to characterize the differences between work life, daily life, and psychological impacts of the COVID-19 pandemic on OTs who work in the physical and mental health fields in medical facilities. Fisher's exact test and two-sample *t*-tests were performed on all items of the online questionnaire and psychological measurements in the fields of physical and mental health. If statistical significance was observed in Fisher's exact test for a questionnaire item with more than three selections, a *post hoc* residual analysis was applied to identify which selection contributed the most to the statistical significance.

In addition, a multinomial logistic regression model for each field was created with psychological measurement (anxiety, depression, insomnia, and loneliness) as independent variables to detect potential factors and subjective quality in one's own and colleagues' therapy services (increased, decreased, and unchanged as a reference variable) as dependent variables, and this model enabled us to test the impact of mental health on the quality of work. In the multinomial logistic regression model, sociodemographic data in each field were transformed into a generalized propensity score, which was used to adjust for potential confounding bias. Variance inflation factor (VIF) was used to check for multicollinearity. All independent variables were allowed places in a multinomial logistic regression model if their VIF values were less than five (35).

The formula is:

Subjective therapy quality in one's own/colleagues' (increased, decreased, and unchanged) ~ SAS score + SDS score + ISI-J score + TILS score + generalized propensity score.

Moreover, generalized linear models (GLMs) were created with the variables that showed statistically significant differences by the field comparison (mental and physical health) in work/daily life as the independent variables and the four psychological measurement scores (SAS, SDS, ISI-J, and TILS scores) as the dependent variables; this model enabled us to detect relationships between psychological impact and changes in work/daily life. The sociodemographic data in the two fields were transformed into a generalized propensity score, which was used to adjust for potential confounding bias in these models; VIF value ≤ 5 was also applied to avoid multicollinearity.

The formula is:

Each psychological measurement score (SAS, SDS, ISI-J, or TILS scores) ~ work/daily life items differed between mental and physical health fields + generalized propensity score.

The results were presented as odds ratios (ORs) or regression coefficients (RC) with 95% confidence intervals (CIs), and the level of statistical significance was set at *p* < 0.05 (two-tailed). All statistical analyses were performed using SPSS (version 25.0; IBM Corp., Armonk, NY, USA).

## Results

### Sample Characteristics and Questionnaire Results

#### Sample Characteristics and Daily Life

**Figure 1:**
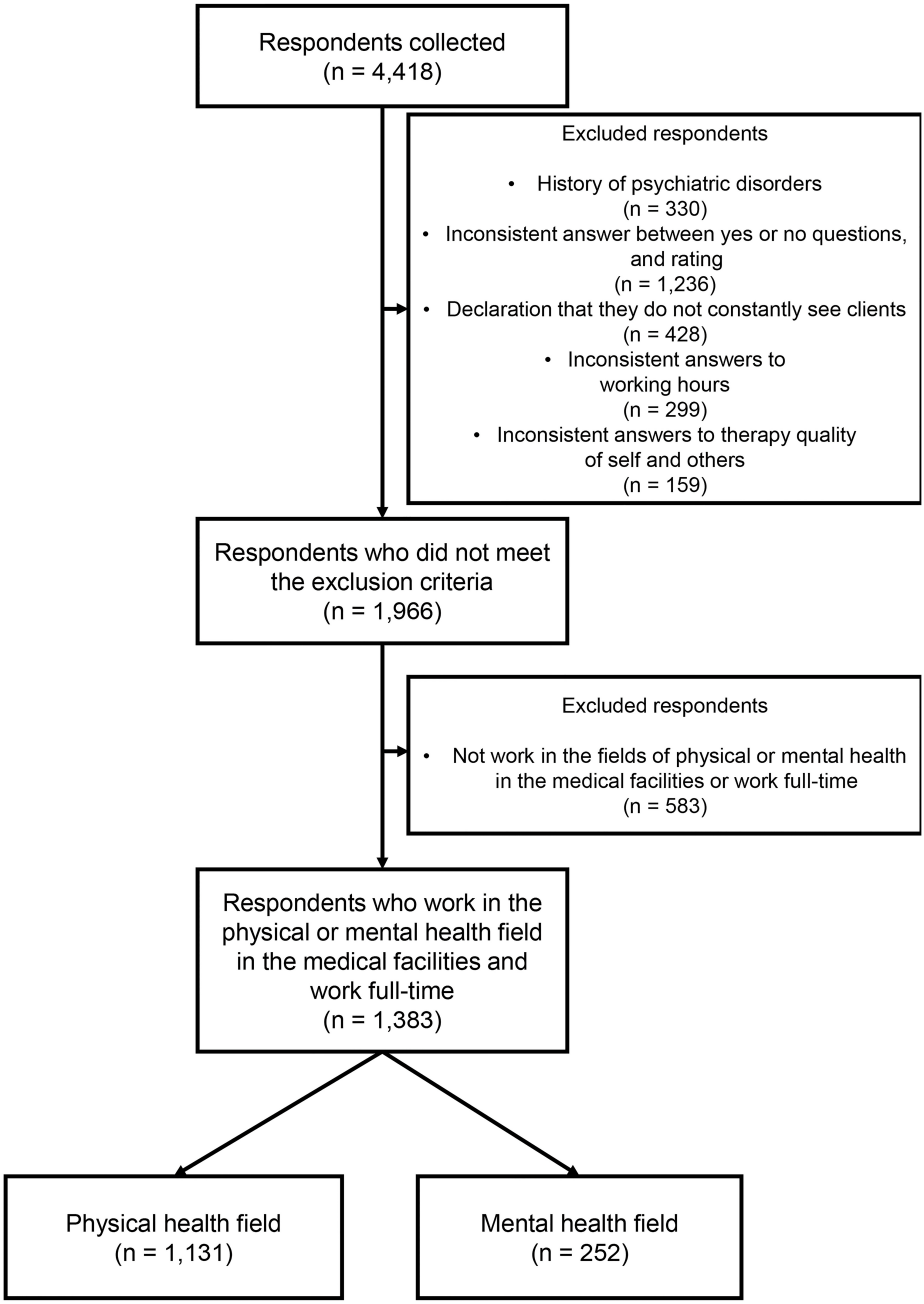
Flow diagram for the respondents included in data analysis. Flow chart summarizing the number of respondents excluded with implementation of each eligibility criteria culminating in the final analytical data set (*n* = 1,383, OTs in the physical health field: *n* = 1,131; OTs in the mental health field: *n* = 252).

The total number of initial respondents was 4,418. To determine data eligibility, the following procedure was used to select the respondents in line with these criteria (Figure 1). First, data from respondents with a history of psychiatric disorders (*n* = 330), inconsistent answers between “yes” or “no” questions and rating (e.g., “yes” to the change in outing frequency but rated the frequency as “unchanged”) (*n* = 1,236), declaration that they do not constantly see clients (*n* = 428), inconsistent answers to items about working hours (*n* = 299), and inconsistent answers to therapy quality of self and others (*n* = 159) were excluded (see Figure 1). The number of remaining respondents was 1,966. Of these respondents, 1,383 of whom worked full-time and in the fields of physical or mental health in medical facilities were identified and classified into two groups: OTs in the physical health field (*n* = 1,131) and OTs in the mental health field (*n* = 252).

Table 1 shows the characteristics and questionnaire results of all participants and those in each of the two health fields. In the sociodemographic data, OTs in the physical health field showed lower values in mean age and service years than OTs in the mental health field [mean age (SD): 35.1 (8.6) years vs. 39.1 (8.3) years, *t* = −6.59, *p* < 0.001; mean service years 11.5 (7.9) year vs. 14.8 (7.7) year, *t* = −6.08, *p* < 0.001]. In addition, a significant difference was observed regarding managerial position, indicating a lower rate of managerial position of OTs in the physical health field compared to those in the mental health field [Fisher's exact test: 351 (31.0%) vs. 106 (42.1%), *p* = 0.001, see Table 1].

**Table 1:** Sample characteristics and questionnaire results regarding daily life.

	**No./Total no. (%)**			
**Characteristics**	**Total sample (*n* = 1,383)**	**OTs in the physical health field (*n* = 1,131)**	**OTs in the mental health field (*n* = 252)**	***p***
**Sample characteristics**				
Mean age (year) (SD)	35.8 (8.7)	35.1 (8.6)	39.1 (8.3)	<0.001*
Sex				0.888
Female	771 (55.7)	629 (55.6)	142 (56.3)	
Male	612 (44.3)	502 (44.4)	110 (43.7)	
Academic background				0.162
< Bachelor	762 (55.1)	613 (54.2)	149 (59.1)	
≥ Bachelor	621 (44.9)	518 (45.8)	103 (40.9)	
Marital status				0.001
Married	840 (60.7)	664 (58.7)	176 (69.8)	
Unmarried	543(39.3)	467 (41.3)	76 (30.2)	
Managerial position				0.001
Yes	457 (33.0)	351 (31.0)	106 (42.1)	
No	926 (67.0)	780 (69.0)	146 (57.9)	
Service years, mean (SD)	12.1 (7.9)	11.5 (7.9)	14.8 (7.7)	<0.001*
**Daily life**				
Efforts to avoid being infected (1 = *never*, 7 = *frequent*)				0.870
5–7	1,370 (99.1)	1,118 (98.9)	252 (100)	
1–3	0 (0.0)	0 (0.0)	0 (0.0)	
4	13 (0.9)	13 (1.1)	0 (0.0)	
Efforts to not transmit the virus to others (1 = *never*, 7 = *frequent*)				0.256
5–7	1,363 (98.5)	1,116 (98.7)	247 (98.0)	
1–3	4 (0.3)	2 (0.2)	2 (0.8)	
4	16 (1.2)	13 (1.1)	3 (1.2)	
Frequency of contact with family (1 = *never*, 7 = *frequent*)				0.983
5–7	940 (68.0)	768 (67.9)	172 (68.3)	
1–3	196 (14.2)	160 (14.1)	36 (14.3)	
4	247 (17.8)	203 (18.0)	44 (17.4)	
Frequency of contact with friends (1 = *never*, 7 = *frequent*)				0.324
5–7	373 (26.8)	302 (26.7)	69 (27.4)	
1–3	637 (46.1)	513 (45.4)	124 (49.2)	
4	375 (27.1)	316 (27.9)	59 (23.4)	
Fewer outings				1.000
Yes	1,365 (98.7)	1,116 (98.7)	249 (98.8)	
No	18 (1.3)	15 (1.3)	3 (1.2)	
Avoidance of face-to-face conversations				0.193
Yes	1,275 (92.2)	1,048 (92.7)	227 (90.1)	
No	108 (7.8)	83 (7.3)	25 (9.9)	
Increased precautions at home				0.393
Yes	1,323 (95.7)	1,079 (95.4)	244 (96.8)	
No	60 (4.3)	52 (4.6)	8 (3.2)	
Increased mask-wearing				0.700
Yes	1,373 (99.3)	1,122 (99.2)	251 (99.6)	
No	10 (0.7)	9 (0.8)	1 (0.4)	
Increased SNS usage				0.889
Yes	709 (51.3)	581(51.4)	128 (50.8)	
No	674 (48.7)	550 (48.6)	124 (49.2)	
Free description about changes in life (fill-in-the-blank question)				0.634
Yes	220 (15.9)	183 (16.2)	37 (14.7)	
No	1,163 (84.1)	948 (83.8)	215 (85.3)	

*SNS, Social Networking Service*.

* *Two-sample t-test*;

† *Fisher's exact test*.

No significant differences were observed between the two groups for any of the items about their daily lives.

#### Therapy Quality, Psychological Measurements, and Work Life

Table 2 shows the questionnaire results of therapy quality, psychological measurements, and work life in OTs who work in the two health fields. Regarding therapy quality, significantly higher ratios of decrease were shown in changes in one's own and colleagues' therapy quality in the mental health field than in the physical health field [*post hoc* residual analysis: 239 (21.1%) vs. 98 (38.9%), *p* < 0.001, and 231 (20.1%) and 94 (37.3%), *p* < 0.001, respectively].

**Table 2:** Questionnaire results of therapy quality, psychological measurements, and work life.

	**No./Total no. (%)**			
**Characteristics**	**Total sample (*n* = 1,383)**	**OTs in the physical health field (*n* = 1,131)**	**OTs in the mental health field (*n* = 252)**	***p***
**Therapy quality**				
Changes in own therapy quality compared with early 2019 (before COVID-19)				<0.001^†^
Increased	91 (6.6)	75 (6.6)	16 (6.3)	1.000^‡^
Decreased	337 (24.4)	239 (21.1)	98 (38.9)	<0.001^‡^
Unchanged	955 (69.0)	817 (72.3)	138 (54.8)	<0.001^‡^
Changes in colleagues' therapy quality compared with early 2019 (before COVID-19)				<0.001^†^
Increased	81 (5.9)	65 (5.8)	16 (6.4)	1.000^‡^
Decreased	325 (23.5)	231 (20.4)	94 (37.3)	<0.001^‡^
Unchanged	977 (70.6)	835 (73.8)	142 (56.3)	<0.001^‡^
**Psychologic measurements**
Presence of anxiety, depression, insomnia, and loneliness (cutoff score)				
SAS (≥ 40)	202 (14.6)	173 (15.3)	29 (11.5)	0.139^†^
SDS (≥ 50)	242 (17.5)	206 (18.2)	36 (14.3)	0.143^†^
ISI-J (≥ 10)	203 (14.6)	168 (14.9)	35 (13.9)	0.768^†^
TILS (≥ 6)	340 (24.6)	275 (24.3)	65 (25.8)	0.628^†^
Raw score on each questionnaire				
SAS	33.6 (6.5)	33.7 (6.6)	32.9 (6.2)	0.067*
SDS	40.6 (8.8)	40 0.7(8.9)	39.9 (8.0)	0.184*
ISI-J	5.3 (4.0)	5.4 (3.9)	5.2 (4.0)	0.652*
TILS	4.3 (1.5)	4.3 (1.5)	4.3 (4.5)	0.560*
**Work life**				
Accepting patients with COVID-19				<0.001^†^
Yes	501 (36.2)	451 (39.9)	50 (19.8)	
No	882 (63.8)	680 (60.1)	202 (80.2)	
Provision of information on COVID-19 by workplace (1 = *never*, 7 = *sufficient*)				0.257^†^
5–7 (above average level)	1,028 (74.3)	846 (74.8)	182 (72.2)	
1–3 (below average level)	132 (9.6)	101 (8.9)	31 (12.3)	
4	223 (16.1)	184 (16.3)	39 (15.5)	
Changes in workload compared with early 2019 (before COVID-19)				<0.001^†^
Increased	660 (47.7)	517 (45.7)	143 (56.7)	0.005^‡^
Decreased	262 (19.0)	218 (19.3)	44 (17.5)	1.000^‡^
Unchanged	461 (33.3)	396 (35.0)	65 (25.8)	0.015^‡^
Changes in working hours compared with early 2019 (before COVID-19)				<0.001^†^
Increased	121 (8.8)	102 (9.0)	19 (7.5)	1.000^‡^
Decreased	133 (9.6)	121 (10.7)	12 (4.8)	0.012^‡^
Unchanged	1,129 (81.6)	908 (80.3)	221 (87.7)	0.017^‡^
Change in homework compared with early 2019				0.491^†^
Increased	9 (0.7)	7 (0.6)	2 (0.8)	
Decreased	79 (5.7)	59 (5.2)	20 (7.9)	
Unchanged	1,295 (93.6)	1,065 (94.2)	230 (91.3)	
Free description about changes in work style (fill-in-the-blank question)				0.919^†^
Yes	233 (16.9)	190 (16.8)	43 (17.1)	
No	1,150 (83.1)	941 (83.2)	209 (82.9)	

*SAS, Zung Self-Rating Anxiety Scale; SDS, Zung Self-Rating Depression Scale; ISI-J, Japanese version of the Insomnia Severity Index; LS, TILS, Japanese version of the three-item loneliness scale*.

* *Two-sample t-test*;

† *Fisher's exact test*;

‡ *Post hoc residual analysis (corrected p-value)*.

In work life, a lower acceptance ratio was found in the mental health field than in the physical health field [Fisher's exact test: 451 (39.9%) vs. 50 (19.8%), *p* <0 0.001]. Additionally, a higher rate of increase and a lower rate of unchanged workload, and a lower ratio of decrease and a higher ratio of unchanged working hours were observed in the mental health field than in the physical health field [*post hoc* residual analysis: increased workload, 517 (45.7%) vs. 143 (56.7%), corrected *p* = 0.005; unchanged workload, 218 (19.3%) vs. 44 (17.5%), corrected *p* = 0.015]; decreased working hours, 121 (10.7%) vs. 12 (4.8%), corrected *p* = 0.012; unchanged working hours, 908 (80.3%) vs. 221 (87.7%), corrected *p* = 0.017.

In psychological measurements, no significant differences between these two fields were observed for any of the items.

### Psychological Factors Impacting Therapy Quality in Each Field

Multinomial logistic regression analyses were performed to examine the psychological impact on changes in therapy quality (own and colleagues) in each field. In these analyses, all the values of VIF are less than five, showing that there is no multicollinearity among the four independent variables (SAS, SDS, ISI-J and TILS; all, VIF ≤ 2.561).

Table 3 shows the results of the multinomial logistic regression analysis for each field. Decrease and increase in therapy quality were significantly associated with SDS (decrease: OR = 1.03, 95% CI [1.00–1.06], *p* = 0.033; increase: OR = 0.96, 95% CI [0.92, 1.00], *p* = 0.043, respectively), and a decrease in colleagues' therapy quality was significantly associated with SDS (decrease: OR = 1.05, 95% CI [1.02–1.08], *p* < 0.001) in the physical health field. In the mental health field, only a decrease in colleagues' therapy quality was significantly associated with the ISI-J (OR = 1.22, 95% CI [1.04–1.44], *p* = 0.015). No significant differences were observed in any of the other psychological measurements that contributed to therapy quality in each field.

**Table 3:** Multinominal logistic regression results predicting psychological impacts on quality of treatment among occupational therapists.

	**Decrease**					**Increase**				
			**95% CI**					**95% CI**		
**Variables**		**Odds ratio**	**Lower**	**Upper**	**p**		**Odds ratio**	**Lower**	**Upper**	**p**
**Physical health field (*****n*** **=** **1,131)**										
**Therapy quality (self)**	***n*** **= 239**					**n = 75**				
Psychological measurement	(Ref. *n* = 817)									
SAS		1.004	0.971	1.039	0.815		1.011	0.956	1.07	0.703
SDS		**1.030**	**1.002**	**1.058**	**0.033**		**0.956**	**0.916**	**0.999**	**0.043**
ISI		1.029	0.958	1.074	0.197		1.081	0.998	1.161	0.056
TILS		1.034	0.93	1.149	0.541		0.904	0.741	1.105	0.325
**Therapy quality (colleague)**	***n*** **= 231**					***n*** **= 65**				
Psychological measurement	(Ref. *n* = 835)									
SAS		0.971	0.938	1.006	0.105		0.993	0.935	1.054	0.806
SDS		**1.049**	**1.020**	**1.079**	**<0.001**		0.984	0.940	1.03	0.491
ISI		1.027	0.983	1.073	0.235		1.018	0.941	1.101	0.657
TILS		1.026	0.921	1.143	0.807		1.025	0.843	1.245	0.807
**Mental health field (n = 252)**										
**Therapy quality (self)**	***n*** **= 98**					**n = 16**				
Psychological measurement	(Ref. *n* = 138)									
SAS		0.902	0.783	1.038	0.815		1.005	0.946	1.068	0.867
SDS		0.983	0.886	1.089	0.149		0.999	0.952	1.048	0.953
ISI		**1.222**	**1.039**	**1.437**	**0.015**		1.024	0.944	1.111	0.571
TILS		0.797	0.476	1.337	0.390		1.119	0.910	1.376	0.285
**Therapy quality (colleague)**	***n*** **= 94**					***n*** **= 16**				
Psychological measurement	(Ref. *n* = 142)									
SAS		1.030	0.969	1.095	0.343		0.985	0.863	1.126	0.829
SDS		0.994	0.947	1.043	0.801		0.940	0.850	1.040	0.233
ISI		1.016	0.937	1.102	0.683		1.154	0.964	1.351	0.174
TILS		1.092	0.888	1.342	0.404		0.885	0.543	1.441	0.623

*This model simultaneously entered psychological measurements as independent variables and standardized propensity scores as covariates. The reference variable as the dependent variable was “Unchanged”. Bold values indicate statistical significance*.

*Ref., reference variable; SAS, Zung Self-Rating Anxiety Scale; SDS, Zung Self-Rating Depression Scale; ISI-J, Japanese version of the Insomnia Severity Index; TILS, Japanese version of the three-item loneliness scale; CI, Confidence Interval*.

### Influence of Work Life Problems on Psychological Measurements

GLM analyses were performed separately to examine the effects of changes in workload and working hours on psychological measurements (see Table 4). In these analyses, the values of VIF are less than five, showing that there is no multicollinearity between the two independent variables (workload and working hours; all, VIF ≤ 1.120). Increased workload was positively associated with anxiety (RC = 0.802, 95% CI [0.277–1.326], *p* = 0.003), depression (RC = 1.840, 95% CI [0.964–2.716], *p* < 0.001), and insomnia (RC = 2.330, 95% CI [1.180–3.481], *p* < 0.001) in OTs in the physical health field, and positively associated with insomnia (RC = 2.453, 95% CI [0.149–4.758], *p* = 0.037) in OTs in the mental health field. Moreover, increased working hours were commonly associated with anxiety in both fields (physical health: RC = 1.566, 95% CI [0.743–2.389], *p* < 0.001; mental health: RC = 3.184, 95% CI [1.342–5.026], *p* < 0.001, respectively).

**Table 4:** Generalized linear model (GLM) for impact of workload and working hours on psychological measurements among occupational therapists.

	**Physical health field (*****n*** **= 252)**						**Mental health field (*****n*** **= 252)**					
				**95% CI**						**95% CI**		
**Variables**	***N***	**Coefficient**	**SE**	**Lower**	**Upper**	***p***	***N***	**Coefficient**	**SE**	**Lower**	**Upper**	***p***
**Anxiety (SAS)**												
*Workload*												
Increased	517	**0.802**	**0.268**	**0.277**	**1.326**	**0.003**	143	0.947	0.598	−0.225	2.119	0.113
Decreased	218	0.329	0.345	−0.347	1.004	0.341	44	−0.022	0.772	−1.536	1.492	0.977
Unchanged (Ref.)	396						65					
*Working hours*												
Increased	102	**1.566**	**0.420**	**0.743**	**2.389**	**<0.001**	19	**3.184**	**0.940**	**1.342**	**5.026**	**0.001**
Decreased	121	−0.169	0.396	−0.945	0.608	0.670	12	−0.627	1.172	−2.924	1.670	0.593
Unchanged (Ref.)	908						221					
**Depression (SDS)**												
*Workload*												
Increased	517	**1.840**	**0.447**	**0.964**	**2.716**	**<0.001**	143	1.062	0.912	−0.725	2.849	0.244
Decreased	218	0.996	0.577	−0.135	2.127	0.084	44	−1.241	1.203	−3.598	1.116	0.302
Unchanged (Ref.)	396						65					
*Working hours*												
Increased	102	0.791	0.701	−0.584	2.165	0.260	19	1.094	1.469	−1.785	3.972	0.457
Decreased	121	−0.561	0.663	−1.860	0.738	0.397	12	−2.421	1.840	−6.029	1.186	0.188
Unchanged (Ref.)	908						221					
**Insomnia (LS)**												
*Workload*												
Increased	517	**2.330**	**0.587**	**1.180**	**3.481**	**<0.001**	143	**2.453**	**1.176**	**0.149**	**4.758**	**0.037**
Decreased	218	0.839	0.757	−0.645	2.323	0.268	44	−0.229	1.545	−3.257	2.799	0.882
Unchanged (Ref.)	396						65					
*Working hours*												
Increased	102	0.516	0.922	−1.291	2.323	0.576	19	2.153	1.874	−1.519	5.825	0.251
Decreased	121	−0.150	0.869	−1.853	1.553	0.863	12	−1.564	2.352	−6.174	3.045	0.506
Unchanged (Ref.)	908						221					
**Loneliness (TILS)**												
*Workload*												
Increased	517	0.195	0.105	−0.011	0.401	0.064	143	0.167	0.221	−0.265	0.600	0.449
Decreased	218	0.153	0.136	−0.114	0.420	0.261	44	0.267	0.288	−0.297	0.831	0.353
Unchanged (Ref.)	396						65					
*Working hours*												
Increased	102	0.054	0.165	−0.270	0.378	0.743	19	0.208	0.348	−0.474	0.890	0.549
Decreased	121	0.232	0.156	−0.074	0.537	0.137	12	0.389	0.436	−0.465	1.243	0.372
Unchanged (Ref.)	908						221					

*This model simultaneously entered workload and working hours as independent variables and standardized propensity scores as covariates. Bold values indicate statistical significance*.

*SE, Standard error; Ref., Reference variable; SAS, Zung Self-Rating Anxiety Scale; SDS, Zung Self-Rating Depression Scale; ISI-J, Japanese version of the Insomnia Severity Index; TILS, Japanese version of the three-item loneliness scale; CI, Confidence Interval*.

## Discussion

To our knowledge, this is the first study to investigate the differences in work life problems between the physical and mental health fields in occupational therapy with a large sample size, and their psychological risk factors as affected by the COVID-19 outbreaks. Overall, 14.6, 17.5, 14.6, and 24.6% of the Japanese OTs involved in this study presented symptoms of anxiety, depression, insomnia, and loneliness, respectively (Table 1). An increase in negative psychological impacts was observed compared to the results of our previous survey conducted between May 28 to May 31, 2020 (6), especially with respect to anxiety and depression (11.3 and 10.6%, respectively, see Table 2). Additionally, in terms of work life, accepting patients with COVID-19 (36.2%) and increased workload (47.7%) and working hours demonstrated a substantial increase compared to our previous report (16.6, 28.5, and 3.4%, respectively). These results support the previous study that elevated psychological distress among healthcare workers was significantly greater during repeated outbreaks, and that longer exposure to psychological distress leads to poor functional outcomes at home and work, heightens the risk of mental health issues and its overt symptoms, and increases healthcare use (10). The results also suggest that OTs are continuously required to take prompt measures for mental health prevention and promotion at the workplace during repeated outbreaks of COVID-19, consistent with findings from a previous global survey by the WFOT (17).

Notably, the differences in work life between the two fields were mainly observed in therapy quality, increased workload, and work time. Despite a lower rate of accepting patients, a greater decrease in one's own and colleagues' therapy quality and increase in workload, and a lower rate of decrease in working hours were observed in the mental health field compared to the physical health field. One of the reasons for these results can be attributed to different work environments. In the mental health field, typical occupational therapy programs target the acquisition of psychosocial benefits through group-based interventions (20, 22). With repeated outbreaks of COVID-19 rendering group activities difficult, not only group therapy targeting multiple patients, but also recreational therapy formed by multidisciplinary cooperation has been severely limited. These factors may have obliged increased efforts among OTs to develop alternative interventions to promote continuity of service delivery for all users, in addition to basic infection prevention and control, resulting in decreased therapy quality and increased workload and work time. Another possible reason is the increased patient vulnerability to a higher risk of infection and mortality due to symptom characteristics of mental illness (e.g., cognitive impairment, limited awareness of risk, and inadequate/diminished efforts regarding personal protection among patients) (27, 36, 37). A previous study reported a seven-fold increase in infection risk of COVID-19 in patients with mental disorders than those without mental disorders (depression: adjusted odds ratio (AOR) controlling demographics, AOR = 10.43, 95% CI [10.10, 10.76]; schizophrenia: AOR = 9.89, 95% CI [8.68–11.26]); bipolar disorder: AOR = 7.69, 95% CI [7.05–8.40] (27). In addition, a previous study investigating the work environment of psychiatric healthcare workers reported a continuously worsening working environment and increased work-related stress during the COVID-19 pandemic in the psychiatric field (38). Another possible explanation is the long length of hospitalization of patients with mental disorders, which is unique to Japan. Psychiatric care in Japan lags behind other countries in terms of deinstitutionalization (39, 40), and a lag of ~266 days was reported in 2018 (41), which is conspicuously longer than that in other Organization for Economic Co-operation and Development (OECD) countries. Nevertheless, no significant difference was observed in any of the psychological measurements (presence rate of symptoms and raw scores).

Additionally, psychological factors in each field were extracted to investigate the impact of therapy quality using a multinomial logistic regression model. These results suggest that the different impacts of psychological conditions in the two domains did affect therapy quality; depression was the main cause of decreased therapy quality in the physical health field, and insomnia was the main cause of decreased therapy quality in the mental health field (Table 3). However, while the differences in therapy quality, workload, and working time between these two fields were expected to readily reveal more apparent psychological problems in the mental health field, these problems were not evident.

Furthermore, the GLM showed a relationship between mental health deterioration and work life, workload, and working hours (Table 4). As a result, increased workload was detected as an important factor in anxiety, depression, and insomnia, and increased working hours were detected in anxiety in the physical health field. In the mental health field, important factors identified were increased working hours for anxiety and increased workload in insomnia. Once again, the relationship between these variables in the mental health field was less pronounced than those in the physical health field.

The relationship between psychological measurements (including anxiety and depression) and workload was found to be significant only in the physical health field, perhaps because other factors might be influencing anxiety and depression in the mental health field. Another reason might be a specific form of social desirability bias. OTs in the mental health field routinely evaluate patients' mental health conditions using these psychological measurements. Therefore, it is possible that they may have estimated their own mental health assessment too high, to portray themselves as ideal therapists who care for patients with mental health problems (42). Another possibility is that they may have acquired effective preventive strategies such as self-care practices, mindsets and avoiding exposing themselves to negative information (9), to mitigate the deterioration of their mental states owing to their high expertise and skills exercised throughout their working lives. Future studies should clarify the coping skills of therapists in the mental health field.

Another possible factor that may cause the difference between the physical interpretation of these findings is that negative mental health conditions of OTs in the mental health field, which are not currently apparent, may gradually or rapidly deteriorate owing to the decreasing quality of treatment as well as increased workload and more working hours. This may be regarded as a finding that anticipates an OT crisis in the mental health and welfare field soon. If this is the case, it may be useful to examine the mental health condition of OTs in the mental health field, especially concerning depression symptoms, and to adapt the environmental setting; this would include facilitating increased staffing, reassignment, the effective use of telerehabilitation (enabling equal patient satisfaction and clinical improvement compared to conventional face-to-face rehabilitation programs) (43, 44), improvement of workplace infrastructure, the adoption of appropriate and shared anti-contagion measures (9). Reduced opportunities for resourcefulness have led to a burden on therapists, opportunities which could prevent the higher risk of anticipated depression symptoms.

This study has several limitations. First, it was conducted using a cross-sectional online questionnaire and focused only on OTs in the physical and mental health fields in Japan. As each of these two fields can have a different working style and healthcare systems can vary across nations, the generalization of the present findings should be carefully considered. Further studies should recruit OTs worldwide to determine whether these results are unique to OTs in Japan and examine whether the present results can be replicated among other second-line workers. Second, OTs in the mental health field could be affected by social desirability biases. In other words, they may overestimate their own mental health assessment. Adding welltrained interviewers and physiological indices that reflect psychological stress states which are less susceptible to these effects would give a clearer picture. Third, this study did not explore the details of each work life problem. Further research should focus on specific work life problems and collect detailed and specific information on aspects such as the type and degree of deterioration in therapy quality and increase in workload, to develop tailored preventive and intervention strategies for field-specific problems. Finally, it should be noted that the present study did not fully capture the influence of COVID-19 on OTs in the two fields examined. To address these issues, we believe that validation of free comments on individual mental health impacts and measures is needed, using the method of a recent study (14). Thus, we recommend that as much support as possible be rapidly afforded to the two groups of OTs.

In conclusion, this study demonstrated the differences in COVID-19 impacts between OTs in the physical and mental health fields, focusing on quality of treatment, psychological condition, and work life. Moreover, the relationships between psychological factors and treatment quality varied across fields. These results reveal the psychological impact of changes in work life due to COVID-19 differed by specialty, even among the same healthcare professionals; *depression* was the main cause of decreased therapy quality in the physical health field, and *insomnia* was the main cause of decreased therapy quality in the mental health field. Thus, we need to investigate the field-specific negative impacts of COVID-19 on OTs as an important step towards devising tailored and effective prevention and intervention strategies. Finally, we believe that the present study makes a significant contribution to the emerging literature on mental health management in the COVID-19 pandemic.

## Data Availability Statement

The original contributions presented in the study are included in the article/supplementary material, further inquiries can be directed to the corresponding authors.

## Ethics Statement

The studies involving human participants were reviewed and approved by Ethics Committee of Saitama Prefectural University. The patients/participants provided their written informed consent to participate in this study.

## Author Contributions

DS, AI, HY, HN, and TI: study conception, design, and data acquisition. DS, AI, and TI: analysis, interpretation of data, and writing—review & editing. DS: writing—original draft. All authors approved final version of the article.

## Funding

This research was supported by the Saitama Prefectural University Grant (200070).

## Conflict of Interest

The authors declare that the research was conducted in the absence of any commercial or financial relationships that could be construed as a potential conflict of interest.

## Publisher's Note

All claims expressed in this article are solely those of the authors and do not necessarily represent those of their affiliated organizations, or those of the publisher, the editors and the reviewers. Any product that may be evaluated in this article, or claim that may be made by its manufacturer, is not guaranteed or endorsed by the publisher.

## Abbreviations

AOR: Adjusted odds ratio
CI: Confidence interval
GLM: Generalized linear model
ISI–J: Japanese version of the Insomnia Severity Index
LS: Japanese version of the three-item loneliness scale
OECD: Organization for Economic Co-operation and Development
OR: Odds ratio
OTs: Occupational therapists
RC: Regression coefficient
SAS: Zung Self-Rating Anxiety Scale
SDS: Zung Self-Rating Depression Scale
WFOT: World Federation of Occupational Therapists
WHO: World Health Organization.
